# Burnout among medical students of the University of Kerbala and its correlates

**DOI:** 10.1186/s43045-021-00152-2

**Published:** 2021-12-17

**Authors:** Marwa Saad Yahya, Ali Abdulridha Abutiheen, Amer Fadhil Al- Haidary

**Affiliations:** 1 Alhur PHC Sector, Kerbala Health Directorate, Kerbala, Iraq; 2 Department of Family and Community Medicine, College of Medicine, University of Kerbala, Kerbala, Iraq; 3 Department of Psychiatry, College of Medicine, University of Kerbala, Kerbala, Iraq

**Keywords:** Burnout, Medical students, Emotional exhaustion, Cynicism, Professional efficacy

## Abstract

**Background:**

Burnout is prevalent among medical students. Throughout their training, medical students face many psychosocial stresses that, if not managed, might cause burnout syndrome. Burnout could negatively impact students’ life and their academic performance. This study aims to estimate the prevalence of burnout among medical students at the College of Medicine, University of Kerbala, and assess factors associated with it.

An analytic cross-sectional study. A sample of 424 students from all academic years from the College of Medicine, University of Kerbala, was approached. Data collection was conducted from June 11 to July 3, 2020, through a self-administered online questionnaire based on the Maslach Burnout Inventory Student Survey (MBI-SS). The MBI-SS included 15 questions with a 7-point rating ranging from 0 (never) to 6 (always). The tool measures three subscales: emotional exhaustion (5 questions), Cynicism (4 questions), and professional efficacy (6 questions). Statistical Package for the Social Sciences (SPSS) program version 24 was used for data analysis. Binary logistic regression was used to assess the association between burnout and students̓ variables, *P* value of a level < 0.05 considered statistically significant.

**Results:**

The prevalence of burnout syndrome among medical students was 38.2%. About 85.6% of students had high emotional exhaustion, 77.8% had high cynicism, and 32.5% exhibited low professional efficacy. Female gender, regular use of legal substances, and family history of mental diseases were associated with significantly high rates of burnout.

**Conclusions:**

Burnout is prevalent among medical students of the University of Kerbala with quite high levels of emotional exhaustion and cynicism and lower professional efficacy levels. Faculties of medicine need to consider burnout among their students and works to reduce unnecessary stresses by modifying and upgrading the educational and clinical environments.

## Background

Burnout currently is a mental condition, that attains growing attention. It was firstly introduced by psychologists Herbert Freudenberger and Christina Maslach in the mid-1970s to describe the experience of being physically and emotionally drained as a result of extended stress. Kafry and Pines were the first to describe student burnout; they defined it as a syndrome characterized by a loss of interest in studying, a lack of motivation, and exhaustion. Student burnout refers to a feeling of exhaustion caused by study pressures, a cynical and detached behavior toward one's studies, and a sense of inadequacy as a student [1–4].

The prevalence of student burnout varies significantly across different settings and geographical regions. Burnout is estimated to impact at least 50% of all medical students during their school, according to US research (included 7 medical schools in the United States) [5, 6]. A systematic review and meta-analysis study (included the following countries USA, Germany, other European countries, Brazil, Australia, and Sweden) showed a range of 7% to 75.2% of burnout rates among medical students. As medical students are frequently exposed to psychosocial stresses where medical training is considered to have an elevated psychological burden. However, stress, burnout, depression, exhaustion, and quality of life, both mental and physical, could all be forms of medical student distress. Maltreatment and improper dealing with medical students may aggravate burnout [6–9].

The increased years of learning, multiple negative life events, stress, feelings of having limited control over life, poor peer relationships and support, lower exercise levels, smoking cigarettes, gender, and increased alcohol consumption were all associated with burnout among medical students [10–12].

Early detection of burnout is an effective strategy for preventing and/or reducing the burnout impacts, such as exhaustion, incredulity, and a sense of poor professional efficacy, and probably students’ academic performance. And medical colleges must assure that they have adequate support and interventions for students who may experience greater stress and emotional discomfort during their studies [13–15]. No previous studies in Iraq on burnout have been carried out before, especially in medical students, So this study aimed to estimate the prevalence of burnout among medical students in KMC and the factors associated with it. We hypothesized that the prevalence of burnout in our sample would be similar to that described in the literature (50%) [5, 6].

## Methods

An analytical cross-sectional study was conducted at the College of Medicine, University of Kerbala, Kerbala, Iraq. The participants were medical students from all 6 stages.

Kerbala Medical College (KMC) included 1016 medical students from 6 years of study.

Due to the coronavirus COVID-2019 pandemic situation, most universities shifted to online classes around the world [16]. So, the data collection was conducted through an online approach using over the period from June 11 to July 3, 2020.

According to the following formula for determination of sample size *n* = *Z*^2^ *P* (1-*p*)/*d*^2^ , *n* = sample size, *Z* = 1.96 which is the corresponding value for the 95% confidence interval, *P* = prevalence of burnout among medical students, *d* = the degree of precision was at 0.05 at 95% confidence interval, and anticipated prevalence of burnout among medical students = 50%

$$\mathrm{Sample}\;\mathrm{size}=\frac{\left(1.96\right)^2\ast0.05\ast\left(1-0.5\right)}{\left(0.05\right)^2}=\frac{0.9515}{0.0025}=380$$

Supposing the non-response rate, 10% of the sample size was added, so, the minimum required sample size was 380+38 =418 students.

A convenient sample of 424 students from the total number of 1016 medical students at the year 2019–2020 (628 pre-clinical and 388 clinical) was approached in the study. Thus, the response rate was 42%.

A self-administered online questionnaire using Google form was developed. Then it was distributed as a link to the student representatives of each stage who shared it with all (1016) other students through the telegram groups which were already formed for each stage academic year and requesting their voluntary participation. Email survey took about 3 weeks to complete. The email survey took about 5–8 min for one participant to be completed.

### Inclusion criteria

Inclusion criteria include all of the undergraduate medical students including both gender (male and female) and 18 years or above, who are willing to participate in the study and to complete all items of the MBI-SS.

The questionnaire consisted of 2 parts, the first part contains socio-demographic characteristics like gender, age, marital status, residency, academic stage, family income, smoking habit and type of smoking, exercise habit, daily sleep hours, regular use of legal substances (pain relieve drugs, sedatives, cough drugs, steroids, antidepressants, caffeine), and family history of psychological disorders. Where to maintain maximum health, adults should sleep 7 or more hours every night regularly (according to the American Academy of Sleep Medicine and Sleep Research Society). And regular exercise can improve anxiousness, tension, and a general sense of well-being [17, 18].

The second part of the questionnaire included Maslach Burnout Inventory -Student Survey (MBI-SS). The Arabic version was previously used and validated by other studies. It is a modified version of the Maslach Burnout Inventory-General Survey (MBI-GS), which had been adjusted for use on students and become the classically used tool to measure academic burnout syndrome [19, 20].

The MBI-SS includes 15 questions that conform to three scales: Emotional Exhaustion (5 questions), Cynicism (4 questions), and Professional Efficacy (6 questions). The MBI-SS questions are scored by students on a 7-point scale, based on their indicated frequency, ranging from 0 to 6, where 0, never; 1, a few times a year; 2, monthly; 3, a few times a month; 4, weekly; 5, a few times a week; and 6, every day [21].

The internal consistency for the MBI-SS total 15 questions was 0.76. The internal consistency for the subscales were 0.75 (Cronbach’s *a* = 0.75) for the emotional exhaustion 5 questions, 0.8 (Cronbach’s *a* = 0.8) for the cynicism 4 questions, and 0.73 (Cronbach’s *a* = 0.73) for the professional efficacy 6 questions.

The authors used a similar classification of other studies for burnout diagnosis. Students were identified as exhibiting burnout (high level) if their scores were more than 14 for emotional exhaustion and cynicism (score >6) while the low for professional efficacy (score < 23). These results matched the 66th percentile of exhaustion and cynicism, as well as the 33rd percentile of efficacy. Low burnout level indicated emotional exhaustion (< 10) cynicism (< 2), and efficacy (scores > 27). A moderate level of burnout was identified according to the following scores: emotional exhaustion (10–14), cynicism (2–6), and professional efficacy (23–27) [8, 22].

The study is a part of research that was approved by the Research Ethics Committee of the College of Medicine, University of Kerbala. The participation was entirely voluntary and anonymous, the participants were able to respond at their convenience, and the confidentiality of the answers was ensured.

Data analysis was done using the Statistical Package for Social Sciences (SPSS) version 24 computer software; categorical variables were presented as frequencies and percentages. Quantitative variables were expressed as mean and standard deviation (SD). Binary logistic regression had been used to evaluate the association between burnout and students̓ background variables. All predictor variables were tested in one block to assess their predictive ability. Adjusted odds ratios (AOR) and 95% confidence intervals (95% CI) were measured. A *p* value of less than 0.05 was considered statistically significant.

## Results

### Description of the sample

About 145 students (34.2%) were within the age group ≥22 years, 295 (69.6%) of them were females, 402 (94.8%) were singles, 313 (73.8%) were from Kerbala, and 392 (92.5%) were nonsmokers.

Other characteristics are shown in Table 1.

**Table 1:** Socio-demographic characteristics of students from the College of Medicine, University of Kerbala (*N*=424)

Characteristics		Number ***N*** = 424	Percentage %
**Age/years**	18–19	142	33.5
**Mean ±SD** (20.74±2)	20–21	137	32.3
**Range** (18_33)	≥22	145	34.2
**Gender**	Male	129	30.4
	Female	295	69.6
**Marital status**	Single	402	94.8
	Married	19	4.5
	Separated/divorced	3	0.7
**Residence**	Kerbala	313	73.8
	Others	111	26.2
**Stage of study**	First	163	38.4
	Second	62	14.6
	Third	58	13.7
	Fourth	57	13.4
	Fifth	35	8.3
	Sixth	49	11.6
**Smoking status**	Smoker	30	7.0
	Non-smoker	392	92.5
	Ex-smoker	2	0.5
**Exercise habit per week**	Never	63	15
	Rarely	158	37.3
	Some times	170	40.1
	Always	33	7.8
**Daily Sleep hours**	<7 h	106	25.0
	≥7 h	318	75.0
**Regular use of legal substances**^a^	Yes	56	13.2
	No	368	86.8
**Family history of mental diseases**	Yes	36	8.5
	No	388	91.5

^a^Regular use of legal substances included (pain relief drugs, sedatives, cough drugs, steroids, antidepressants, caffeine)

### Prevalence of burnout syndrome

Emotional exhaustion, cynicism, and professional efficacy categories had been used to classify burnout scales. As a result of this classification, 85.6% of students were classified as having high emotional exhaustion, 77.8% as having high cynicism, and 32.5% as exhibiting low professional efficacy as shown in Table 2.

**Table 2:** Prevalence of burnout syndrome among students from the College of Medicine, University of Kerbala, according to the subscales

Burnout syndrome subscales and levels with scores	Number ***N***=424	Percent (%)
**MBI emotional exhaustion**		
Low (0–9)	24	5.7%
Moderate (10–14)	37	8.7%
High (>14)	363	85.6%
**MBI cynicism**		
Low (0–1)	31	7.3%
Moderate (2–6)	63	14.9%
High (>6)	330	77.8%
**Low professional efficacy**		
Low (>27)	193	45.5%
Moderate (23–27)	93	22 %
High (<23)	138	32.5%

According to the three-dimensional criterion of burnout, the burnout diagnosis was calculated as a combination of the 3 sub-domains (high exhaustion + high cynicism + low professional efficacy); 162 of the students (38.2%) were experiencing burnout, while 262 (61.80%) students had no burnout.

Using binary logistic regression to find the association between socio-demographic variables and burnout syndrome, after adjusting for multiple variables in this analysis, burnout was associated with (*P* value < 0.05): female gender (adjusted OR= 1.824, 95% CI 1.074–3.095), regular use of legal substances (adjusted OR= 1.868, 95% CI 1.001–3.484), and family history of mental diseases (adjusted OR= 2.65 95% CI 1.251–5.624). Other variables did not show significant association with burnout syndrome as shown in Table 3.

**Table 3:** Association between three-dimensional burnout diagnosis and socio-demographic characteristics of students from the College of Medicine, University of Kerbala (*N*=424)

Variables		Adjusted OR	OR CI 95% Lower-Upper	***P*** value
**Age groups/years**	18–19	2.058	0.697–6.072	0.191
	20–21	1.319	0.570–3.055	0.518
	≥22 (ref.)	1		
**Gender**	Male (ref.)	1		
	Female	1.824	1.074–3.095	**0.026**
**Marital status**	Single	0.943	0.080–11.135	0.963
	Married	0.439	0.029–6.584	0.552
	Separated/divorced (ref.)	1		
**Residence**	Kerbala	1.023	0.616–1.696	0.931
	Others (ref.)	1		
**Smoking status**	Smoker	0.670	0.030–15.184	0.802
	Non-smoker	0.278	0.013–5.983	0.414
	Ex-smoker (ref.)	1		
**Stage**	First	0.741	0.218–2.519	0.631
	Second	0.855	0.260–2.810	0.796
	Third	1.047	0.355–3.088	0.934
	Fourth	1.461	0.6–3.561	0.404
	Fifth	2.334	0.908–6	0.079
	Sixth (ref.)	1		
**Exercise habit**/**week**	Never	1.541	0.611–3.163	0.360
	Rarely	1.265	0.553–2.897	0.578
	Sometimes	0.80	0.353–1.893	0.598
	Always (ref.)	1		
**Daily sleep hours**	<7	1.456	0.879–2.413	0.145
	≥7 (ref.)	1		
**Regular use of legal substances**^a^	Yes	1.868	1.001–3.484	**0.05**
	No (ref.)	1		
**Family history of mental diseases**	Yes	2.653	1.251–5.624	**0.011**
	No (ref.)	1		

^a^Regular use of legal substances included (pain relief drugs, sedatives, cough drugs, steroids, antidepressants, caffeine)

## Discussion

Medical students are more likely than non-medical students to have physical and mental health problems. As medical college is a demanding educational environment where learners are constantly required to acquire and recall a lot of knowledge in a fixed timeframe [16, 23]. In Iraq, there are a few studies that had assessed the stress among medical students [24]. Up to our knowledge, this study is probably the first study to cover this important mental hazard and focusing the sight on burnout among medical students.

According to the three-dimensional criteria of the MBI-SS, more than one-third (38.2%) of medical students have burnout. However, a variant range of burnout rates among medical students from different countries. The finding of the current study matched the previous study conducted by Shadid et al. in Saudi Arabia showed that 38.2% of the medical students exhibited a high burnout rate [25]. While lower rates of burnout among medical students were reported in other studies performed by Altannir et al. (from Saudi Arabia) and Almeida et al. (from Brazil), represented 13.4% and 14.9%, respectively [23, 26].

The high burnout level could reflect a stressful teaching environment in KMC and other medical colleges in Iraq, and perhaps this is a reality. However, other explanations for such differences in burnout rates might be related to variant instruments used to evaluate burnout among medical students. Further, some studies used the two-dimensional criteria, which were less delicate than those used in the current study (three-dimensional criteria). Furthermore, students’ psychological stress and burnout were considered to be increased by online learning. Thus, the curfew and the necessity accelerated shift to online teaching due to the COVID-19 pandemic could be a part of increasing burnout levels among medical students at the University of Kerbala [8, 27, 28].

When each dimension of burnout was assessed separately, medical students from the University of Kerbala had high percentages of emotional exhaustion (85.6%) and cynicism (77.8%), but lower percentages in reduced professional efficacy (32.5%). Similar findings were described by other studies [8, 26], which reported high rates of cynicism and emotional exhaustion, while lower rates in decreased professional efficacy. This implies that in medical students, a high degree of professional effectiveness can compensate for the stress of academic life. While students with a high level of burnout had lower academic efficacy [29].

However, these results differ from the results of Altannir et al., who indicated lower levels of emotional exhaustion, high cynicism, and high levels of reduced professional efficacy [24].

The difference in these results between the current study and the previously mentioned study may be due to different instruments and cutoff points used to calculate burnout (risk calculation methods). The MBI Questionnaire that was used in Altannir et al.’s study was a 22-item instrument with the following scores for burnout diagnosis: emotional exhaustion ≥30, cynicism ≥12, and professional efficacy ≤33 [24], in addition to the variations in curricula between medical colleges and differences in the demography of medical students from one country to another.

Binary logistic regression analysis, reveals that three variables were associated with Burnout; gender (female), medications intake, history of mental disorders within the family, and the number of study days per week ≤ 2.

The significantly higher percentages of burnout among female students are agreed with other studies done in Saudi Arabia, Pakistan, India (Kerala), Ethiopia (Debre Berhan), and Croatia [11, 30–34], which reported that female gender was associated with high burnout rates than male. In contrast, other studies in Thailand and Brazil reported high burnout rates among males as compared with females medical students [8, 35]. While other studies reported no significant gender differences in burnout rates [2, 36, 37]. However, it is documented that females and female medical students are more vulnerable to stress and depression, general anxiety which can be contributed to their high rates of burnout as compared to male students [30, 38].

The regular use of legal substances among medical students was significantly among medical students was significantly related to high burnout rates in this study. Also, a significant relationship between the occurrence of burnout and drug intake was reported in a study done in Brazil among dental students [39].

While other studies were done in Hong Kong and Srinagar, had reported no association between regular use of legal sunstances and burnout [40, 41]. The Regular use of legal substances among students could be a part of a particular disease (physical or psychological) that might have a negative impact on students’ psych and as a consequence, resulting in a higher risk for burnout.

The current study showed that the presence of mental diseases within the family can predict for burnout of medical students. While a study done in Nepal showed no association between family history of mental health problems and burnout among medical students [42]. The students whose relatives had mental health conditions might take on the responsibility of caretaker for them. Thus, those students might expose to daily tension in the context of caring for individuals with mental disorders and thus can lead to burnout. A positive family history of mental diseases might provide a genetic vulnerability in reaction to stressful events (evidence for gene-stress interaction) [43].

### Limitation

The limitations of this study include the non-probability sample of the students, due to the online approach. So probably, those who get interested in the subject had participated, and those who do not participate could have certain viewpoints differ than the participant. The study was planned to be on a random sample through direct contact, but the emergent COVID-19 pandemic changes the hall academic year and turned most of it to online learning. Similarly, the study plan changed to an online survey. However, this shift might give light to the shift to the electronic learning effect. Another limitation is that this study does not measure the impact of burnout on the academic performance of students.

## Conclusions

This study identifies that a considerable prevalence (more than one-third) of medical students of the University of Kerbala had burnout, with quite high rates of emotional exhaustion and cynicism, and a low level of professional efficacy. Burnout was significantly associated with female gender, regular use of legal substances, and family history of mental diseases. Faculty must focus on altering improving the academic and clinical conditions, context, and curricula to reduce unnecessary stresses and create more favorable teaching and clinical practice. As well as that professors need to understand the students’ needs, motivations, and experiences to keep students interested in education and to minimize their burnout rates.

## Data Availability

Data available on request from the corresponding author.

## Abbreviations

KMC: Kerbala Medical College
PBL: Problem-based approach learning
MBI-GS: The Maslach Burnout Inventory-General Survey
MBI-SS: The Maslach Burnout Inventory-Student Survey
